# Autonomic Function and Baroreflex Control in COVID-19 Patients Admitted to the Intensive Care Unit

**DOI:** 10.3390/jcm13082228

**Published:** 2024-04-12

**Authors:** Francesca Gelpi, Maddalena Alessandra Wu, Vlasta Bari, Beatrice Cairo, Beatrice De Maria, Tommaso Fossali, Riccardo Colombo, Alberto Porta

**Affiliations:** 1 Department of Biomedical Sciences for Health, University of Milan, 20133 Milan, Italy; francesca.gelpi@unimi.it (F.G.); beatrice.cairo@unimi.it (B.C.); alberto.porta@unimi.it (A.P.); 2 Department of Biomedical and Clinical Sciences, University of Milan, 20157 Milan, Italy; maddalena.ale.wu@gmail.com; 3 Division of Internal Medicine, ASST Fatebenefratelli Sacco, Luigi Sacco Hospital, 20157 Milan, Italy; 4 Department of Cardiothoracic, Vascular Anaesthesia and Intensive Care, IRCCS Policlinico San Donato, 20097 Milan, Italy; 5 IRCCS Istituti Clinici Scientifici Maugeri, 20138 Milan, Italy; beatrice.demaria@icsmaugeri.it; 6 Department of Anesthesiology and Intensive Care, ASST Fatebenefratelli-Sacco, Luigi Sacco Hospital, 20157 Milan, Italy; tommaso.fossali@asst-fbf-sacco.it (T.F.); riccardo.colombo@asst-fbf-sacco.it (R.C.)

**Keywords:** SARS-CoV-2, heart rate variability, arterial blood pressure, autonomic nervous system, cardiovascular regulation, respiratory failure, modified head-up tilt

## Abstract

**Background**: Autonomic function and baroreflex control might influence the survival rate of coronavirus disease 2019 (COVID-19) patients admitted to the intensive care unit (ICU) compared to respiratory failure patients without COVID-19 (non-COVID-19). This study describes physiological control mechanisms in critically ill COVID-19 patients admitted to the ICU in comparison to non-COVID-19 individuals with the aim of improving stratification of mortality risk. **Methods**: We evaluated autonomic and baroreflex control markers extracted from heart period (HP) and systolic arterial pressure (SAP) variability acquired at rest in the supine position (REST) and during a modified head-up tilt (MHUT) in 17 COVID-19 patients (age: 63 ± 10 years, 14 men) and 33 non-COVID-19 patients (age: 60 ± 12 years, 23 men) during their ICU stays. Patients were categorized as survivors (SURVs) or non-survivors (non-SURVs). **Results**: We found that COVID-19 and non-COVID-19 populations exhibited similar vagal and sympathetic control markers; however, non-COVID-19 individuals featured a smaller baroreflex sensitivity and an unexpected reduction in the HP-SAP association during the MHUT compared to the COVID-19 group. Nevertheless, none of the markers of the autonomic and baroreflex functions could distinguish SURVs from non-SURVs in either population. **Conclusions**: We concluded that COVID-19 patients exhibited a more preserved baroreflex control compared to non-COVID-19 individuals, even though this information is ineffective in stratifying mortality risk.

## 1. Introduction

As severe acute respiratory syndrome coronavirus 2 (SARS-CoV-2) spread worldwide in early 2020, critically ill patients with coronavirus disease 2019 (COVID-19) pneumonia emerged as a primary challenge in intensive care units (ICUs) [1,2,3]. A hallmark of COVID-19 severity is a hyperinflammatory immune response accompanied by multi-organ dysfunction [1,2,3,4,5,6,7,8,9]. Sudden respiratory failure, necessitating mechanical ventilation, is often associated with disseminated coagulopathy and hemodynamic instability [2,3,5,6]. The typical dysregulated immune response leads to an uncontrolled hyperinflammation referred to as “cytokine storm” [4,9,10,11].

The autonomic nervous system (ANS) oversees all unconscious physiological processes. The vagal branch of the ANS is an important modulator of the inflammatory pathway and can also enhance anti-viral immunity [8,12]. When the vagal activity is low, the anti-inflammatory response might be weak, contributing to the cytokine storm [8,11]. Furthermore, the hyper-immune reaction associated with COVID-19 produces a significant adrenergic release that is only partially modulated by the activity of sympathetic nerves [11]. In addition to inducing modifications in the activity of the ANS, SARS-CoV-2 viral infection harms structures of the ANS directly [13] and indirectly via immune cell infiltration into the central nervous system [14]. The cardiac arm of the baroreflex is a regulatory reflex aiming at limiting short-term variability of arterial pressure via modifications of heart rate [15]. Since baroreflex is a mainly vagal reflex [15], it is not surprising that the COVID-19-associated sympathetic activation [16] keeps the baroreflex function low [17].

Modifications of the autonomic control and baroreflex function following COVID-19 infection have often been studied via the analysis of spontaneous changes in heart period (HP) and systolic arterial pressure (SAP) [17,18]. Remarkably, it was found that the magnitude of HP changes holds prognostic value in critically ill COVID-19 patients [1,7,8,19,20]. Lower values of HP variability were associated with the severity of COVID-19 [19], while higher values were a distinctive feature of surviving COVID-19 patients admitted to the ICU [1]. Moreover, smaller changes in HP were reported to precede the increase in inflammatory markers [7], to predict ICU indication and admission in the first week after hospitalization [8] and to characterize patients who received mechanical ventilation [20]. Survivors of mild COVID-19 at 3–6 months exhibited an impaired baroreflex response that might explain the incidence of postural hypotensive episodes in post-COVID-19 patients [17]. Robust applications of HP variability markers require a challenge to probe cardiovascular control [21], the monitoring of breathing rate to ensure its consistency across groups and experimental conditions [22,23], the analysis of spontaneous fluctuations of variables that are more sensitive to sympathetic control (such as the SAP [24,25]) and the assessment of baroreflex [26].

The clinical value of autonomic and baroreflex control markers derived from spontaneous variations of physiological variables in stratifying the mortality risk in ICU patients without COVID-19 (non-COVID-19) is well-recognized [21,27,28,29]. However, it is unknown whether the same markers could differentiate COVID-19 and non-COVID-19 individuals and could be fruitfully used to stratify mortality risk within the COVID-19 group.

Thus, the aim of this study is to compare autonomic function and baroreflex regulation in non-COVID-19 and COVID-19 patients admitted to the ICU for respiratory failure and to assess the ability of these markers in stratifying the mortality risk in the two groups. Autonomic and baroreflex control indexes were derived from HP and SAP variability while monitoring the ventilatory rate [21]. Cardiovascular regulatory mechanisms were probed via a validated challenge, namely the modified head-up tilt (MHUT) [30], utilized in the ICU setting to disturb homeostasis by inducing sympathetic activation in response to central hypovolemia [21]. Preliminary results were presented at the 2022 Computing in Cardiology conference [31].

## 2. Materials and Methods

### 2.1. Experimental Protocol

From September 2020, we consecutively enrolled 17 critically ill COVID-19 patients (min-max range, age: 43–79 years, body mass index: 23–33 kg·m^−2^; 14 men) and 33 critically ill non-COVID-19 patients (min-max range, age: 32–74 years, body mass index: 20–43 kg·m^−2^; 23 men). Both populations were admitted to the ICU with a diagnosis of respiratory failure, defined as the need for mechanical ventilation or continuous positive airway pressure by helmet, because of pneumonia or acute respiratory distress syndrome. COVID-19 was defined as a positive real-time polymerase chain reaction for SARS-CoV-2 on nasal swabs or bronchoalveolar lavage fluid. The study was performed according to the Declaration of Helsinki in regard to medical research involving humans. The study was approved by the ethical review board of the “L. Sacco” Hospital, Milan, Italy, namely the Comitato Etico Interaziendale Area 1, Milan, Italy (protocol number 13465 on 22 December 2011) and its amendment during the COVID-19 pandemic (approval number 2020/ST/116 on 14 May 2020). Protocol was registered at ClinicalTrials.gov (NCT01930669 on 3 November 2014). All conscious patients gave their written informed consent, while close relatives or legal representatives provided the written consent on behalf of unconscious patients. Exclusion criteria included being aged below 18 years, the significant presence of non-sinus cardiac beats (>5%), spinal or head injuries, suspected or documented intracranial hypertension and contraindications of any kind to postural modifications.

A surface electrocardiogram (ECG) and invasive arterial pressure (AP) were acquired from the patients’ monitors in the ICU (IntelliVue MX800 Patient Monitor, Philips, Best, The Netherlands). Signals were sampled at 500 Hz, and Vital Recorder software (version 1.9.1.0) [32] was utilized to collect signals from the monitor. Experimental sessions took place during each patient’s first day in the ICU. The signals were recorded at rest in the supine position (REST) for 10 min followed by an additional 10 min during MHUT. The MHUT maneuver was applied by exploiting the standard ICU three-segment bed (Total Care, Hill-Rom Company, Batesville, IN, USA). The ability of the MHUT to probe autonomic function and baroreflex control was investigated in [30] and its use in the ICU was validated in [21]. Briefly, the MHUT maneuver was performed as follows: the patients’ bed was first tilted to 15° as a rigid body, then the inclination of the back rest was increased to reach 60°, while the thigh rest was adjusted to 0° and the shank rest was left to 15°. All patients were able to complete the overall protocol without experiencing any sign of pre-syncope. All mechanically ventilated patients underwent protective ventilation in volume-control mode at 6–8 mL·kg^−1^. The patients were classified as survivors (SURVs) and non-survivors (non-SURVs) according to the in-hospital patient’s outcome starting from his/her admission to the ICU.

### 2.2. Extraction of Beat-to-Beat Variability Series

After detecting the QRS complex on the ECG, HP was computed as the time interval between two consecutive R-wave peaks. The maximum of AP within the current HP was taken as the current SAP. The amplitude of the first R-wave delimiting the current HP, taken from the isoelectric line to the R-wave apex, was exploited to monitor respiration (R) [33]. The HP, SAP and R values were monitored on a beat-to-beat basis. The resulting variability series were manually corrected in the case of missing beats or misdetections. If non-sinus beats were present, HP, SAP and R measures were linearly interpolated using the closest values unaffected by non-sinus beats. Corrections did not exceed 5% of the total sequence length. Sequences of 256 consecutive HP, SAP and R values were randomly selected within the REST and MHUT sessions. In the time domain, we computed the mean and variance of the HP and SAP series, namely μ_HP_, σ^2^_HP_, μ_SAP_, and σ^2^_SAP_. The indexes were expressed in ms, ms^2^, mmHg and mmHg^2^, respectively.

### 2.3. Frequency Domain Markers of the Autonomic Function

Analysis in the frequency domain was carried out after fitting the series with an autoregressive (AR) model [34]. The model parameters were estimated by solving the least squares identification problem via the Levinson–Durbin recursion [34]. The model order was optimized via the Akaike figure of merit in a range from 8 to 14 [35]. We computed the power of HP in the high frequency (HF) band (i.e., from 0.15 to 0.4 Hz) and the power of SAP in the low frequency (LF) band (i.e., from 0.04 to 0.15 Hz), denoted, respectively, as HF_HP_ and LF_SAP_ and expressed in absolute units (i.e., ms^2^ and mmHg^2^, respectively). HF_HP_ and LF_SAP_ were taken, respectively, as a marker of vagal modulation directed to the heart [36,37] and sympathetic modulation directed to the vessels [24,25]. The frequency of the dominant oscillation of the R series within the HF band was taken as the respiratory frequency (f_R_). f_R_ was expressed in breaths per minute (bpm).

### 2.4. Frequency Domain Indexes of the Baroreflex Control

Baroreflex control was characterized through cross-spectral analysis of the SAP and HP variability series [26,38]. Cross-spectral analysis was carried out via a parametric method grounded on the bivariate AR model with order fixed to 10 [39]. The transfer function modulus from SAP to HP (|H_HP-SAP_(*f*)|) and HP-SAP squared coherence [K^2^_HP-SAP_(*f*)] were computed as a function of the frequency *f* [38].

The averaged |H_HP-SAP_(*f*)| in the LF band, indicated as |H_HP-SAP_(LF)|, was taken as an estimate of baroreflex sensitivity (BRS) in the LF band [40] and labelled as BRS_LF_. BRS_LF_ was expressed in ms·mmHg^−1^. The averaged K^2^_HP-SAP_(*f*) in the LF band was calculated as a marker of the degree of baroreflex engagement [40] and labelled as K^2^_LF_. K^2^_LF_ was dimensionless and ranged between 0 and 1, indicating, respectively, full uncoupling and perfect association between the SAP and HP [38].

### 2.5. Statistical Analysis

Population characteristics were tested according to an χ^2^ test for categorical variables and a Mann–Whitney rank sum test for continuous variables.

Two-way repeated measures analysis of variance (one factor repetition, a Holm–Sidak test for multiple comparisons) was applied to check whether indexes exhibited between-group differences within the same experimental condition (i.e., REST or MHUT) and between-condition differences within the same population (i.e., non-COVID-19 or COVID-19 and SURV or non-SURV).

Statistical analysis was carried out using a commercial statistical program (Sigmaplot v.14.0, Systat Software, San Jose, CA, USA). A *p* < 0.05 was always considered as significant.

## 3. Results

Population characteristics reported in Table 1 showed that non-COVID-19 patients featured a less frequent administration of steroids, a less negative Richmond agitation-sedation scale (RASS score), a shorter length of stay in the ICU, and a lower intra-ICU mortality than COVID-19 patients. The proportion of subjects under mechanical ventilation and modality of ventilation were similar in non-COVID-19 and COVID groups as well as the SOFA score. The higher mortality in the COVID-19 group could be explained by the higher incidence of complications associated with micro and macrothrombosis typical of severe COVID-19.

Table 2 and Table 3 report, respectively, the population characteristics of non-COVID-19 and COVID-19 patients divided into SURVs and non-SURVs. No differences were detected between SURVs and non-SURVs except for the expected higher intra-ICU mortality in non-SURVs compared to SURVs in both the non-COVID-19 and COVID-19 groups.

Table 4 compares univariate HP, SAP and R markers derived from non-COVID-19 and COVID-19 populations at REST and during MHUT. Univariate indexes did not vary during the MHUT compared to REST in either the non-COVID-19 or COVID-19 population. COVID-19 patients exhibited longer μ_HP_ than non-COVID-19 regardless of the experimental condition, as well as lower μ_SAP_ solely at REST.

Table 5 and Table 6 show the univariate HP, SAP and R indexes computed in non-COVID-19 and COVID-19 populations, respectively, divided in SURVs and non-SURVs at REST and during MHUT. No significant differences were detected between groups given the experimental condition (i.e., REST or MHUT) and between experimental conditions given the group (i.e., SURVs or non-SURVs). This result held in both the non-COVID-19 and COVID-19 populations. It is worth noting that, regardless of the experimental condition, non-SURVs had a tendency of exhibiting lower HF_HP_ and higher σ^2^_SAP_ than SURVs in both the non-COVID-19 and COVID-19 populations.

**Figure 1:**
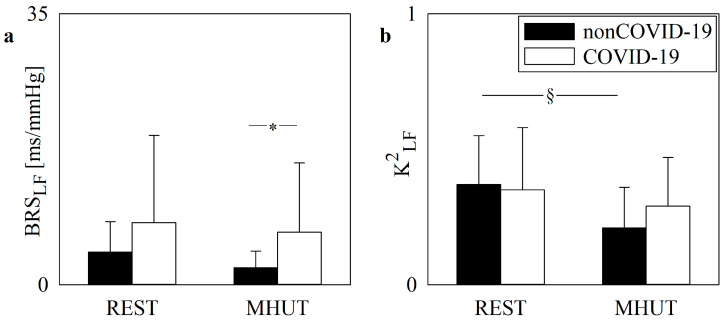
The vertical grouped error bar graphs show the baroreflex control indexes as a function of the experimental condition (i.e., REST and MHUT) in non-COVID-19 (black bars) and COVID-19 (white bars) patients. BRS_LF_ and K^2^_LF_ are reported in (**a**) and (**b**), respectively. The symbol * indicates a significant difference between populations within the same experimental condition with *p* < 0.05, while the symbol § indicates a significant difference between experimental conditions within the same population with *p* < 0.05.

The vertical grouped error bar graphs of Figure 1 show the baroreflex control indexes as a function of the experimental condition (i.e., REST and MHUT) in non-COVID-19 (black bars) and COVID-19 (white bars) populations. BRS_LF_ and K^2^_LF_ are reported in Figure 1a,b, respectively. During the MHUT, BRS_LF_ was higher in COVID-19 patients than in non-COVID-19 patients (Figure 1a), while, in the non-COVID-19 population, K^2^_LF_ decreased significantly during the MHUT compared to REST (Figure 1b).

**Figure 2:**
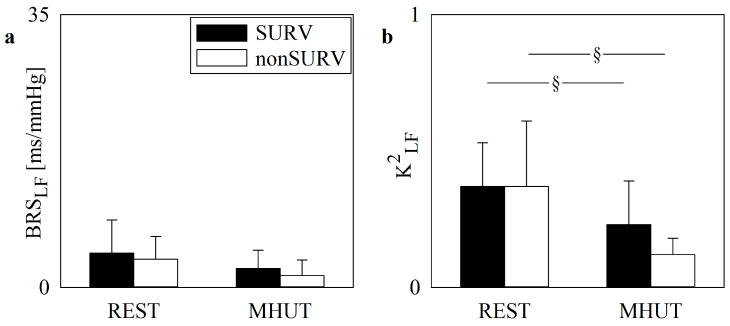
The vertical grouped error bar graphs show the baroreflex control indexes as a function of the experimental condition (i.e., REST and MHUT) in SURV (black bars) and non-SURV (white bars) patients in the non-COVID-19 population. BRS_LF_ and K^2^_LF_ are reported in (**a**) and (**b**), respectively. The symbol § indicates a significant difference between experimental conditions within the same group with *p* < 0.05.

**Figure 3:**
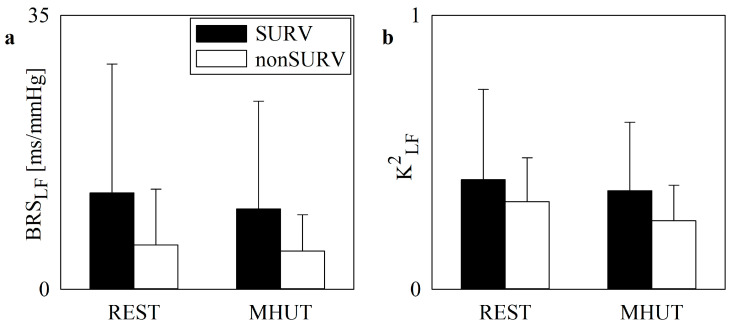
The vertical grouped error bar graphs show the baroreflex control indexes as a function of the experimental condition (i.e., REST and MHUT) in SURV (black bars) and non-SURV (white bars) patients in the COVID-19 population. BRS_LF_ and K^2^_LF_ are reported in (**a**) and (**b**), respectively.

Figure 2 and Figure 3 have the same layout as Figure 1, but they show BRS_LF_ and K^2^_LF_ markers in non-COVID-19 and COVID-19 populations, respectively, categorized into SURVs (black bars) and non-SURVs (white bars). In the non-COVID-19 group, K^2^_LF_ decreased significantly during the MHUT in both SURVs and non-SURVs (Figure 2b), while modifications of BRS_LF_ with the experimental condition were not significant (Figure 2a). In the COVID-19 population, baroreflex control markers did not vary with outcome and experimental condition (Figure 3a,b).

## 4. Discussion

The main findings of this study can be summarized as follows: (i) non-COVID-19 and COVID-19 groups exhibited similar vagal and sympathetic control markers both at REST and during the MHUT, and the orthostatic challenge evoked a negligible autonomic response in both groups; (ii) the impairment of baroreflex control in non-COVID-19 patients took the form of a reduced strength of the HP-SAP coupling during the MHUT compared to REST; (iii) HP-SAP association was more preserved in COVID-19 patients and during the MHUT and BRS_LF_ was higher than in non-COVID-19 patients; (iv) neither autonomic nor baroreflex control markers could differentiate SURVs and non-SURVs, and this result held regardless of the population (i.e., COVID-19 or non-COVID-19 group).

### 4.1. Autonomic Control of Non-COVID-19 and COVID-19 Patients Admitted to the ICU for Respiratory Failure

In our study we compared autonomic control markers derived from the analysis of the HP and SAP variability series in non-COVID-19 and COVID-19 groups admitted to the ICU for respiratory failure. Since pharmacological treatment (such as the administration of catecholamine) and intervention (such as deepness of sedation) were similar, we hypothesize that eventual differences between non-COVID-19 and COVID-19 patients are due to the autonomic control. The COVID-19 and non-COVID-19 groups differed in regard to the administration of steroids; however, since HP variability indexes are expected to be negligibly affected by corticosteroids [41], the impact on conclusions of the administration of the steroids is limited. As a matter of fact, the σ^2^_HP_ and σ^2^_SAP_ did not vary across populations (i.e., non-COVID-19 and COVID-19 groups) and experimental conditions (i.e., REST and MHUT), and this conclusion held even when the markers were made more specific by assessing the magnitude of variations in frequency bands typical of the vagal and sympathetic controls (i.e., HF_HP_ and LF_SAP_) [22,23,24,25,36,37]. Thus, we conclude that the autonomic control of the COVID-19 group is not significantly different from that of the non-COVID-19 group when individuals admitted to the ICU for respiratory failure are considered.

The reduction in the venous return imposed by the orthostatic challenge is expected to evoke sympathetic activation [25,42,43,44], and this finding holds even in the case of an orthostatic challenge of limited intensity such as the MHUT [30]. The missing sympathetic activation in response to the MHUT stresses the limited activity of autonomic control mechanisms in both populations resulting from the combined action of therapeutic interventions in ICU and pathology [21]. Remarkably, the weakness of the response of the autonomic control to the orthostatic challenge is again similar in both groups, thus stressing similarities in autonomic regulations once more.

### 4.2. Baroreflex Control of Non-COVID-19 and COVID-19 Patients Admitted to the ICU for Respiratory Failure

In healthy subjects, the sympathetic activation and vagal withdrawal in response to a postural challenge [25,42,43,44] is accompanied by an augmented engagement of the baroreflex [45,46], as denoted by the rise of K^2^_LF_ [47], and by the decrease in the BRS_LF,_ indicating smaller variations in HP per unit modification of SAP [43,47,48]. In the specific case of the MHUT, it was found that, in healthy subjects, K^2^_LF_ remained unvaried, while BRS was reduced [30]. Conversely, in the non-COVID-19 group, the MHUT reduced the engagement of the cardiac arm of the baroreflex, as denoted by the decrease in K^2^_LF_, thus suggesting an impairment of the baroreflex that appears to be evident when challenged. Since this finding is less evident in COVID-19 patients, we conclude that this reflex is less impaired in the COVID-19 group compared to the non-COVID-19 one. The more efficient cardiac arm of the baroreflex was confirmed by the higher BRS_LF_ during MHUT observed in COVID-19 patients compared to the non-COVID-19 group. Given that the cardiac arm of the baroreflex is a vagal reflex dramatically limited by a complete cholinergic blockade [15], we speculate that vagal control of COVID-19 individuals was more pronounced than in non-COVID-19 individuals. It is unknown whether this phenomenon could be a response to hyperinflammation in the attempt of blunting it in the context of a COVID-19 cytokine storm [1,8,11,12,49]. The marked vagal control in COVID-19 patients may be driven by pulmonary afferences triggered by COVID-19-induced pneumonia. Lung damage by SARS-CoV-2 is characterized by the extensive development of microthrombi and capillary hyperpermeability due to endothelial damage [6,50], thus leading to vascular congestion and extensive extravascular edema, which may activate pulmonary stretch receptors and, consequently, the vagal afferent pathway. Remarkably, the better efficiency of the baroreflex in COVID-19 patients is a finding exclusively associated to their ICU stay given that baroreflex was found to be impaired in SURVs of mild COVID-19 at 3–6 months [17]. The different characteristics of the baroreflex control in the COVID-19 group compared to non-COVID-19 individuals in the ICU was unveiled after the application of the postural challenge [30], thus stressing the importance of probing cardiovascular control in critically ill patients in whom the combination of therapeutic interventions and disease dampens physiological control mechanisms under REST conditions [21].

### 4.3. Association of Autonomic Markers of Non-COVID-19 and COVID-19 Patients Admitted to the ICU for Respiratory Failure with Mortality

In both non-COVID and COVID populations, HF_HP_ and σ^2^_SAP_ were similar in SURVs and non-SURVs, and they were not affected by MHUT. Regardless of the experimental condition, we observed a tendency towards higher values of HF_HP_ in SURVs compared to non-SURVs in both populations, thus suggesting that having a greater vagal modulation might be a protective factor [7,8,19,49]. Regardless of the experimental condition, we observed a tendency of σ^2^_SAP_ towards larger values in non-SURVs compared to SURVs in both populations, thus suggesting that uncontrolled fluctuations of SAP have a negative impact on the survival rate [21,51].

### 4.4. Association of Baroreflex Control Markers of Non-COVID-19 and COVID-19 Patients Admitted to the ICU for Respiratory Failure with Mortality

Given that, during MHUT, the association between the variability of SAP and changes in HP is weak, this study confirms the limited ability of non-COVID-19 patients admitted to the ICU to limit fluctuations of SAP [21] via the activation of the cardiac arm of the baroreflex. However, this limited capability is shared by both SURVs and non-SURVs, being of little help in stratifying the risk of mortality in the non-COVID-19 population. The trend toward a more remarkable decrease in K^2^_LF_ during MHUT in non-SURVs suggests that markers describing the baroreflex control could provide useful indications to stratify the mortality risk in the non-COVID-19 group by highlighting that a worse baroreflex regulation should be considered a negative prognostic factor.

In the COVID-19 group, the impairment of the cardiac arm of the baroreflex control is less evident compared to non-COVID-19 patients, and this behavior is observed in both SURVs and non-SURVs. However, the tendency toward lower BRS_LF_ and K^2^_LF_ both at REST and during MHUT in non-SURVs suggests that a less active baroreflex control might be a negative prognostic factor. We suggest that SURVs within the COVID-19 group might have more powerful internal regulatory resources to limit the progress of inflammation, prevent exaggerated immune responses and avoid the development of arrhythmic events [1,8,11,12,22,49,51]. The more limited activity of the cardiac arm of the baroreflex might contribute to the worse prognosis associated with small HP variations in critically ill COVID-19 patients [1,7,8,19,20].

### 4.5. Limitations of the Study and Future Developments

The limited size of non-COVID-19 and COVID-19 groups is the most remarkable limitation of the study. Increasing the size might improve the statistical power, thus unveiling significant differences that remain undetected in the present study (e.g., the more important decrease in the HP-SAP association during MHUT in non-SURVs, especially in the non-COVID-19 group). The limited association between HP and SAP, especially visible under orthostatic challenges and indicating a more important isolation of the heart and vascular system, could be better explored in future studies via more specific complexity metrics [52,53]. The 70% and 82% of the subjects were males in the non-COVID-19 and COVID-19 groups, respectively. The imbalance is the effect that men are at higher risk of developing respiratory failure compared to women [54]. This disparity might have contributed to bias results of survival analysis in both non-COVID-19 and COVID-19 groups, even though proportions of men were similar in SURVs and non-SURVs. Future studies should clarify whether conclusions might be affected by the gender imbalance of COVID-19 and COVID-19 groups.

## 5. Conclusions

This study compared the autonomic and baroreflex control markers in COVID-19 and non-COVID-19 patients admitted to the ICU for respiratory failure. An orthostatic challenge designed for the application in the ICU, namely the MHUT, was applied to stimulate a response of physiological regulatory mechanisms and unveil any potential impairment in hospitalized patients. Unlike autonomic markers, baroreflex control indexes could differentiate COVID-19 and non-COVID-19 patients. We suggest that baroreflex regulation might be a distinctive feature of COVID-19 patients compared to the non-COVID-19 population. Specifically, the more preserved baroreflex control of COVID-19 individuals points toward a more active vagal control potentially playing an anti-inflammatory role in the context of the hyperinflammatory immune response. While neither autonomic nor baroreflex control indexes were found to be associated with mortality, some indications were provided, suggesting that a more preserved cardiac arm of the baroreflex might contribute to enhancing the likelihood of survival. These insights deserve further exploration, particularly with expanded populations, to better understand their potential in stratifying mortality risk in the ICU.

## Figures and Tables

**Table 1:** Population characteristics of non-COVID-19 and COVID-19 populations.

Variable	Non-COVID-19 (n = 33)	COVID-19 (n = 17)
Age [years]	59.9 ± 11.9	62.5 ± 9.8
Gender [male]	23 (70)	14 (82)
BMI [kg·m^−2^]	27.1 ± 4.9	28.3 ± 2.6
Mechanical ventilation	29 (88)	16 (94)
Administration of catecholamines	17 (52)	8 (46)
Sedation	29 (88)	16 (94)
Steroids	10 (30)	16 (94) *
Septic shock	6 (18)	0 (0)
SOFA score	8.2 ± 3.4	9.5 ± 3.1
RASS score	−3.5 ± 1.6	−4.7 ± 1.2 *
LOS in ICU [days]	10.5 ± 8.1	18.8 ± 12.1 *
Intra-ICU mortality	6 (18)	11 (65) *

BMI = body mass index; SOFA = sequential organ failure assessment; RASS = Richmond agitation-sedation scale; LOS = length of stay; ICU = intensive care unit. Categorical variables are presented as number (percentage). Continuous variables are presented as mean ± standard deviation. The symbol * indicates a significant difference with *p* < 0.05.

**Table 2:** Characteristics of SURVs and non-SURVs within non-COVID-19 population.

Variable	SURV (n = 25)	Non-SURV (n = 8)
Age [years]	61.5 ± 9.9	54.7 ± 15.4
Gender [male]	18 (72)	5 (62)
BMI [kg·m^−2^]	27.9 ± 5.2	24.8 ± 3.3
Mechanical ventilation	21 (84)	8 (100)
Administration of catecholamines	13 (52)	4 (50)
Sedation	21 (84)	8 (100)
Steroids	6 (24)	4 (50)
Septic shock	3 (12)	3 (37)
SOFA score	8.0 ± 3.4	8.6 ± 3.2
RASS score	−3.2 ± 1.7	−4.4 ± 0.9
LOS in ICU [days]	16.1 ± 10.8	8.7 ± 6.1
Intra-ICU mortality	0 (0)	6 (75) *

BMI = body mass index; SOFA = sequential organ failure assessment; RASS = Richmond agitation-sedation scale; LOS = length of stay; ICU = intensive care unit. Categorical variables are presented as number (percentage). Continuous variables are presented as mean ± standard deviation. The symbol * indicates a significant difference with *p* < 0.05.

**Table 3:** Characteristics of SURVs and non-SURVs within the COVID-19 population.

Variable	SURV (n = 6)	Non-SURV (n = 11)
Age [years]	57.2 ± 7.3	65.5 ± 9.8
Gender [male]	6 (100)	8 (73)
BMI [kg·m^−2^]	28.9 ± 2.5	28 ± 2.6
Mechanical ventilation	6 (100)	10 (91)
Administration of catecholamines	3 (50)	5 (45)
Sedation	6 (100)	10 (91)
Steroids	6 (100)	10 (91)
Septic shock	0 (0)	0 (0)
SOFA score	10.3 ± 2.3	9 ± 3.3
RASS score	−4.8 ± 0.2	−4.5 ± 1.4
LOS in ICU [days]	15.7 ± 9.8	20.5 ± 12.9
Intra-ICU mortality	0 (0)	11 (100) *

BMI = body mass index; SOFA = sequential organ failure assessment; RASS = Richmond agitation-sedation scale; LOS = length of stay; ICU = intensive care unit. Categorical variables are presented as number (percentage). Continuous variables are presented as mean ± standard deviation. The symbol * indicates a significant difference with *p* < 0.05.

**Table 4:** Univariate HP, SAP, and R markers within non-COVID-19 and COVID-19 populations.

Variable	Non-COVID-19 (n = 33)		COVID-19 (n = 17)	
	REST	MHUT	REST	MHUT
μ_HP_ [ms]	711 ± 140	696 ± 153	824 ± 184 *	819 ± 171 *
σ^2^_HP_ [ms^2^]	151 ± 245	108 ± 175	169 ± 205	218 ± 309
HF_HP_ [ms^2^]	20 ± 46	21 ± 46	16 ± 30	35 ± 73
μ_SAP_ [mmHg]	127 ± 18	118 ± 18	115 ± 19 *	107 ± 21
σ^2^_SAP_ [mmHg^2^]	11 ± 11	15 ± 14	9 ± 13	14 ± 16
LF_SAP_ [mmHg^2^]	1.8 ± 4.1	0.5 ± 0.9	0.2 ± 0.4	0.5 ± 1.0
*f*_R_ [bpm]	18 ± 4	18 ± 5	18 ± 5	18 ± 4

REST = rest in supine position; MHUT= modified head-up tilt; μ = mean; σ^2^ = variance; HP = heart period; SAP = systolic arterial pressure; μ_HP_ = HP mean; σ^2^_HP_ = HP variance; μ_SAP_ = SAP mean; σ^2^_SAP_ = SAP variance; HF = high frequency; HF_HP_ = HP power in the HF band expressed in absolute units; LF = low frequency; LF_SAP_ = SAP power in the LF band expressed in absolute units; *f*_R_ = respiratory frequency. Variables are presented as mean ± standard deviation. The symbol * indicates a significant difference with *p* < 0.05 compared to the non-COVID-19 group within the same experimental condition.

**Table 5:** Univariate HP, SAP, and R markers of SURVs and non-SURVs in the non-COVID-19 group.

Variable	SURV (n = 25)		Non-SURV (n = 8)	
	REST	MHUT	REST	MHUT
μ_HP_ [ms]	726 ± 148	716 ± 156	666 ± 105	635 ± 135
σ^2^_HP_ [ms^2^]	159 ± 265	115 ± 191	124 ± 183	86 ± 118
HF_HP_ [ms^2^]	23 ± 52	25 ± 51	9 ± 15	10 ± 21
μ_SAP_ [mmHg]	126 ± 19	119 ± 20	130 ± 16	116 ± 14
σ^2^_SAP_ [mmHg^2^]	11 ± 11	13 ± 10	12 ± 13	22 ± 21
LF_SAP_ [mmHg^2^]	1.6 ± 3.9	0.5 ± 0.9	2.5 ± 5.0	0.5 ± 1.0
*f*_R_ [bpm]	18 ± 4	18 ± 5	19 ± 4	19 ± 6

REST = rest in supine position; MHUT= modified head-up tilt; μ = mean; σ^2^ = variance; HP = heart period; SAP = systolic arterial pressure; μ_HP_ = HP mean; σ^2^_HP_ = HP variance; μ_SAP_ = SAP mean; σ^2^_SAP_ = SAP variance; HF = high frequency; HF_HP_ = HP power in the HF band expressed in absolute units; LF = low frequency; LF_SAP_ = SAP power in the LF band expressed in absolute units; *f*_R_ = respiratory frequency. Variables are presented as mean ± standard deviation.

**Table 6:** Univariate HP, SAP, and R markers of SURVs and non-SURVs in COVID-19 group.

Variable	SURV (n = 6)		Non-SURV (n = 11)	
	REST	MHUT	REST	MHUT
μ_HP_ [ms]	844 ± 242	849 ± 242	813 ± 156	803 ± 128
σ^2^_HP_ [ms^2^]	191 ± 218	318 ± 485	157 ± 208	164 ± 161
HF_HP_ [ms^2^]	33 ± 48	69 ± 118	7 ± 7	17 ± 22
μ_SAP_ [mmHg]	106 ± 22	106 ± 22	120 ± 15	108 ± 22
σ^2^_SAP_ [mmHg^2^]	4 ± 4	9 ± 5	11 ± 16	17 ± 19
LF_SAP_ [mmHg^2^]	0.3 ± 0.6	0.2 ± 0.4	0.1 ± 0.2	0.7 ± 1.1
*f*_R_ [bpm]	17 ± 1	17 ± 1	18 ± 5	19 ± 5

REST = rest in supine position; MHUT= modified head-up tilt; μ = mean; σ^2^ = variance; HP = heart period; SAP = systolic arterial pressure; μ_HP_ = HP mean; σ^2^_HP_ = HP variance; μ_SAP_ = SAP mean; σ^2^_SAP_ = SAP variance; HF = high frequency; HF_HP_ = HP power in the HF band expressed in absolute units; LF = low frequency; LF_SAP_ = SAP power in the LF band expressed in absolute units; *f*_R_ = respiratory frequency. Variables are presented as mean ± standard deviation.

## Data Availability

The data presented in this study are available on request from the corresponding author. The data are not publicly available because they contain sensitive personal information.
